# The influence of fluorination on the dynamics of the F^−^ + CH_3_CH_2_I reaction

**DOI:** 10.1039/d3cp02110f

**Published:** 2023-06-21

**Authors:** Thomas Gstir, Tim Michaelsen, Bryan A. Long, András B. Nacsa, Atilay Ayasli, Dasarath Swaraj, Fabio Zappa, Florian Trummer, Shaun G. Ard, Nicholas S. Shuman, Gábor Czakó, Albert A. Viggiano, Roland Wester

**Affiliations:** a Institut für Ionenphysik und Angewandte Physik, Universität Innsbruck 6020 Innsbruck Austria roland.wester@uibk.ac.at; b Air Force Research Laboratory, Space Vehicles Directorate Kirtland Air Force Base NM 87117 USA; c MTA-SZTE Lendület Computational Reaction Dynamics Research Group, Interdisciplinary Excellence Centre and Department of Physical Chemistry and Materials Science, Institute of Chemistry, University of Szeged, Rerrich Béla tér 1 Szeged H-6720 Hungary

## Abstract

The competition between the bimolecular nucleophilic substitution (S_N_2) and base-induced elimination (E2) reaction and their intrinsic reactivity is of key interest in organic chemistry. To investigate the effect of suppressing the E2 pathway on S_N_2 reactivity, we compared the reactions F^−^ + CH_3_CH_2_I and F^−^ + CF_3_CH_2_I. Differential cross-sections have been measured in a crossed-beam setup combined with velocity map imaging, giving insight into the underlying mechanisms of the individual pathways. Additionally, we employed a selected-ion flow tube to obtain reaction rates and high-level *ab initio* computations to characterize the different reaction pathways and product channels. The fluorination of the β-carbon not only suppresses the E2-reaction but opens up additional channels involving the abstraction of fluorine. The overall S_N_2 reactivity is reduced compared to the non-fluorinated iodoethane. This reduction is presumably due to the competition with the highly reactive channels forming FHF^−^ and CF_2_CI^−^.

## Abstract 2

Fluorination at the beta-carbon of ethyl iodide leads to a set of new product channels in the reaction with fluorine anions, which show rich reaction dynamics.

## Graphical abstract

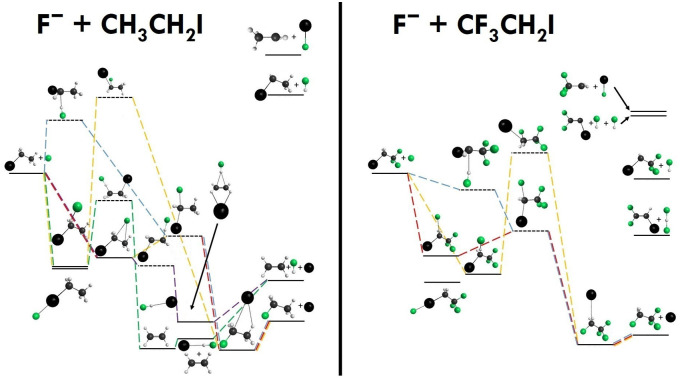

## 1. Introduction

In organic chemistry, the steric properties of a molecule can have a vital influence on its reactivity. Therefore stereospecific reaction pathways are a ubiquitous tool in synthesis processes. In larger molecules, the desired reaction often occurs in competition with others. One archetypal stereospecific reaction is the bimolecular nucleophilic substitution reaction (S_N_2),^1,2^ which has been of scientific interest for over a century.^3,4^ In haloalkanes with multiple carbon centers, another important exchange reaction, base-induced elimination (E2), becomes accessible. The reactivity of S_N_2 and E2 pathways are influenced similarly by the attacking nucleophile/base, the leaving group, and the solvation environment.^5,6^ This leads to their competition in many systems, the simplest ones being halide anions reacting with ethyl halides. The generally accepted assumption is that the competition is mainly controlled by the steric environment of the reaction centers and, thus, their accessibility.^7,8^ Recent theoretical studies, however, have shown that the efficiency of the E2 reaction can lead to a suppression of the S_N_2 channel, even though it has a high intrinsic reactivity.^9^ Gas phase studies under single-collision conditions and the absence of solvation effects provide a model environment to study this competition.^10,11^ They are, however, complicated by both reactions resulting in the same ionic product and can, therefore, not be disentangled using standard mass spectrometric techniques. Different experimental approaches, such as the use of dianions,^12^ detection of the neutral products,^13,14^ secondary reactions of the products,^15^ and sophisticated selection of the neutral reaction partners to favor one of the reaction types^16–21^ have been used to disentangle the competition of S_N_2 and E2 in the gas phase. A different approach is to compare theoretical calculations with experimental results. Recent advances in computational chemistry have made it possible to provide detailed insight into S_N_2 and E2 reactions and shed light on the underlying mechanisms. This has been shown to be successful in the reaction F^−^ + CH_3_CH_2_I^21^ and, more recently, in F^−^ + CH_3_CH_2_Cl.^22^ These two ethyl halides are model reactants for the competition of S_N_2 and E2 as they are among the simplest molecules allowing for both reactions.

In this study, we investigate the influence of fluorination of the β-carbon center on the F^−^ + CH_3_CH_2_I reaction. The E2 reaction occurs with an initial proton abstraction and a subsequent three-body breakup. By substituting the CH_3_-moiety at the β-carbon with a CF_3_ group, the initial hydrogen attack is obstructed. This, in turn, should lead to the suppression of the E2 pathway, making it possible to attribute all product I^−^ to the S_N_2 pathway and obtain a more intimate knowledge of its dynamics.

The introduction of other halides in alkyl halides is known to have an adverse effect on S_N_2 reactivity, with an increasing influence the closer the addition occurs to the active center. Hine and coworkers have shown this effect in additional halogenation of methyl halides^23^ and successive fluorination of the β-carbon center in ethyl iodides.^24^ McBee and coworkers found similar results in fluoroalkyl bromides.^25^

Reactions with the fully β-fluorinated molecule, 1,1,1-trifluoro-2-iodoethane (CF_3_CH_2_I), have gained importance in recent years, as it has a short atmospheric lifetime and is, therefore, a potential substitute for chlorofluorocarbons, which contribute to the depletion of atmospheric ozone.^26,27^

The reactions of fluoride with iodoethane (CH_3_CH_2_I) and 1,1,1-trifluoro-2-iodoethane (CF_3_CH_2_I) in the gas phase were investigated using two experimental methods. The first method comprises a crossed molecular beam setup, where the reaction was studied in a collision energy range from 0.5 to 2.0 eV. There we obtain differential cross-sections from the ion-molecule reactions utilizing 3D velocity map imaging. From these, we can extract the product's velocity-integrated angular- and internal energy distributions in the center-of-mass frame. The reaction with iodoethane was measured earlier on the same experimental setup. These non-fluorinated results have, in part, been previously discussed by Carrascosa *et al.*^21^ For the second experimental method, a selected-ion flow tube apparatus to measure reaction rates and branching ratios was employed. Here, lower collision energies from 0.04 to 0.08 eV were investigated. The experimental methods are supplemented by calculations of stationary points along the minimum energy paths.

The manuscript is organized as follows: in the next section, we present the two employed experimental techniques and our computational methods. In Section 3, we present the results for four different reaction products, followed by a discussion of the results and a concluding section.

## 2. Methods

### 2.1 Crossed-beam experiments

For the reactive scattering experiment, we employed a crossed-beam setup^28^ combined with a velocity map imaging (VMI) spectrometer^29^ based on the original design by Eppink and Parker,^30^ with the capability to resolve the full 3D product ion velocity vector. The whole experiment is pulsed with a repetition rate of 20 Hz. To create the reactant fluorine anion, a 1 : 10 mixture of the precursor NF_3_ and Ar is supersonically expanded between two electrodes, where the ionic species is created in a plasma discharge. The generated ions are then extracted perpendicular to their initial direction by a Wiley-McLaren-type spectrometer and subsequently trapped for 40 ms in an octopole radio-frequency ion trap. There, they are thermalized to room temperature by non-reactive collisions with a helium buffer gas, resulting in a typical kinetic spread of 150 meV FWHM of the reactant ions. The timing of opening and closing of the trap-endcap electrodes is used for mass discrimination, selecting only the ionic species of interest. A potential difference between the trap and the VMI electrode stack is applied to bring the ions to the desired velocity, allowing for variation of the collision energy.

At the center of the stack, the ion beam is crossed at a 60° angle with the neutral beam, produced by supersonically expanding a low concentration of 1,1,1-trifluoro-2-iodoethane seeded in helium. To avoid clustering, the neutral-beam valve is heated to around 330 K. By switching on the VMI electrodes after crossing of the beams, any product ions are extracted perpendicular to the scattering plane and hit the imaging detector after a flight distance of about 65 cm. The imaging stack consists of two multi-channel plates (MCP) in chevron configuration and a phosphor screen. The transverse velocities in the scattering plane can be calculated from the position of impact, which is recorded by a CCD camera. Additionally, the flight time is measured by a photo-multiplier-tube, permitting the calculation of the out-of-plane velocity and the mass of the product ions. Combining the two methods allows for calculating the three-dimensional velocity vector for each product ion.

The recorded three-dimensional differential cross-sections are transformed into the center-of-mass frame and projected on a two-dimensional plane, with the velocity components parallel (*v*_*x*_) and perpendicular (*v*_*r*_) to the collision axis. For the transformation into the center-of-mass frame, the velocities of the ion and neutral beam (ionized by electron impact), together with their spread and angular and spatial distributions, are recorded utilizing 2D velocity map imaging, disregarding the time information.

### 2.2 Selected-ion flow tube experiments

Thermal kinetics for F^−^ + CH_3_CH_2_I and F^−^ + CF_3_CH_2_I have been measured using selected-ion flow tube (SIFT) apparatuses, which have been described previously.^31,32^ Both reactions were studied from 300–500 K (0.04–0.06 eV) using a flowing afterglow-SIFT with a quadrupole mass spectrometer and the F^−^ + CH_3_CH_2_I reaction was also studied from 300–600 K (0.04–0.08 eV) using a SIFT with an electron impact ion source and time-of-flight mass spectrometer. Results were consistent between the two instruments. In the first apparatus, F^−^ ions were produced in a flowing afterglow source by introducing F_2_ (0.5% in He, Linde) into an Ar^+^/e^−^ plasma in a fast helium flow.^33^ F^−^ ions were mass-selected using a quadrupole mass filter and injected *via* a Venturi inlet into a 1 m long, 7 cm diameter, stainless steel reaction flow tube. Helium buffer gas (10 std. L min^−1^), maintained at a pressure of typically 0.5 mbar, carried the ions downstream where the reactant neutral was added in known concentration using a mass flow meter (MKS inc.) through a stainless steel finger inlet 54 cm prior to the end of the flow tube. The flow was sampled through a 2 mm aperture into a high vacuum region where ions were transported using a rectilinear quadrupole ion guide to the entrance of a quadrupole mass spectrometer. Relative ion concentrations were measured as a function of reactant concentration and kinetics derived through standard means.^31^ In the second apparatus, F^−^ ions were produced in an electron impact source by impinging an electron beam of ∼70 eV energy on CF_4_ (Matheson). Mass selection and subsequent reaction were similar to that described above, except that an orthogonally-accelerated time-of-flight mass spectrometer was used for detection.

### 2.3 Computational details

The characterization of the F^−^ + CH_3_CH_2_I stationary points is based on a previous publication,^34^ where the F^−^ + CH_3_CH_2_Cl reaction was analyzed. At the minimum and the transition-state geometries, the Cl is replaced with I and the C–I bond is elongated by approximately 20%. These structures are optimized and harmonic vibrational frequencies are also computed using the second-order Møller–Plesset (MP2) perturbation theory^35^ with the correlation-consistent aug-cc-pVDZ-PP basis set.^36^ The PP stands for pseudo-potential, meaning we replace the inner-core 1s^2^ 2s^2^ 2p^6^ 3s^2^ 3p^6^ 3d^10^ electrons of the iodine with a relativistic effective core potential.^36^ To achieve more accurate energies and frequencies for the newly-found stationary points, we also utilize the explicitly-correlated coupled cluster singles, doubles and perturbative triples CCSD(T)-F12a method^37^ combined with the cc-pVDZ-PP-F12 basis set,^38^ which was directly optimized for explicitly-correlated computations. For the second reaction (F^−^ + CF_3_CH_2_I) we implement a similar process: we substitute the hydrogen atoms on the β-carbon with fluorine atoms and the Cl with an I at the F^−^ + CH_3_CH_2_Cl stationary points, elongate the C–I bond and perform MP2/aug-cc-pVDZ-PP and then CCSD(T)-F12a/cc-pVDZ-PP-F12 geometry optimizations and harmonic vibrational frequency computations. In this case, most of the geometries belonging to the elimination reaction path do not converge. Finally, for both reactions, we obtain classical relative energies, and, considering the zero-point energy corrections, adiabatic relative energies. The *ab initio* computations are performed using the MOLPRO program package.^39^

## 3. Results

**Fig. 1:**
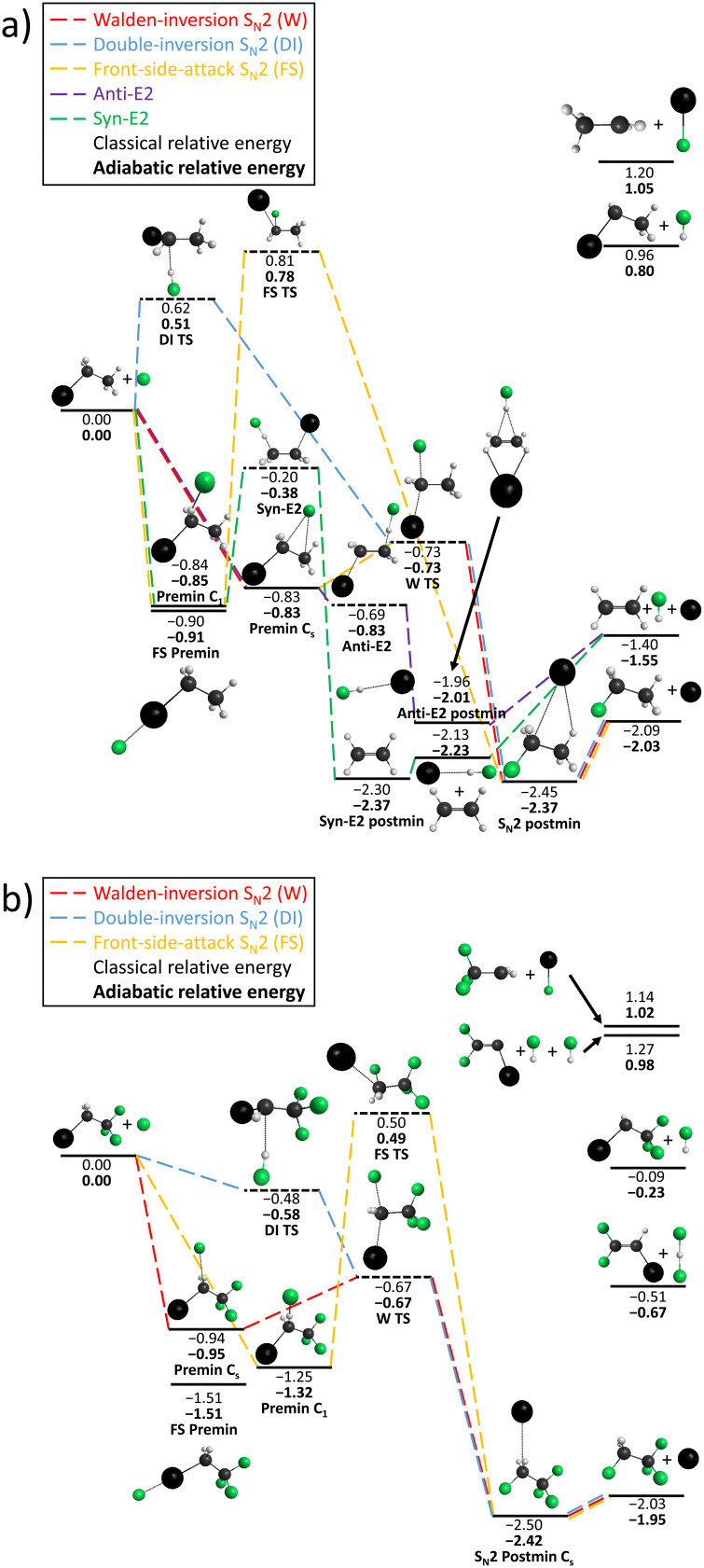
Schematic depiction of the minimum reaction energy pathways for the competing channels E2 and S_N_2 calculated using CCSD(T)-F12a/cc-pVDZ-PP-F12 for (a) F^−^ + CH_3_CH_2_I, where both channels are accessible and for (b) F^−^ + CF_3_CH_2_I, where E2 is passivated. At the transition states and the minima, the geometric arrangements are shown. The dynamics of the individual reaction pathways have been described in detail previously.^11,40,41^ All energy values are given in eV.

Table 1 lists the different reaction pathways for F^−^ + CH_3_CH_2_I and F^−^ + CF_3_CH_2_I observed in the crossed molecular-beam experiment, and in part in the SIFT experiment, in the examined collision energy ranges. Additionally, the calculated reaction enthalpies for the individual channels are given, which are also visualized in Fig. 1. In the SIFT experiment, all exothermic channels, except proton transfer in the reaction with iodoethane, are observed. The endothermicities of the other channels are too high to be overcome even by the maximum collision energy of 0.05 eV. These channels are listed in Table 2, together with their Langevin capture and measured reaction rates.

All observed reaction pathways for the two reactions of fluoride with iodoethane and 1,1,1-trifluoro-2-iodoethane with their respective enthalpies at 0 K, calculated at the CCSD(T)-F12a/cc-pVDZ-PP-F12 level of theory

**Table:** 

Reaction 1	Product	Enthalpy (eV)
F^−^ + CH_3_CH_2_I	→CH_3_CH_2_F + I^−^	−2.03
	→CH_2_CH_2_ + FH + I^−^	−1.55
	→CH_3_CH_2_ + FI^−^	1.05
	→CH_2_CH_2_ + FHI^−^	−2.23
	→FH + CH_3_CHI^−^	0.80

**Table:** 

Reaction 2	Product	Enthalpy (eV)
F^−^ + CF_3_CH_2_I	→CF_2_CHI + FHF^−^	−0.67
	→CF_3_CH_2_F + I^−^	−1.95
	→CF_3_CH_2_ + FI^−^	1.02
	→(FH)_2_ + CF_2_CI^−^	0.98
	→FH + CF_3_CHI^−^	−0.23

Reaction rates and branching factors obtained from the selected-ion flow tube measurements in the collision energy range 0.03 to 0.05 eV for the reactions F + CH_3_CH_2_I and F^−^ + CF_3_CH_2_I. The capture rate constant *k*_cap_ was calculated according to the parametrization by Su and Chesnavich^42^ using *D* = 1.976 D^43^ and *α* = 9.0 Å^3^ for CH_3_CH_2_I and *D* = 1.8 D^43^ and *α* = 8.6 Å^3^ for CF_3_CH_2_I. The polarizability *α* is calculated using the method of Rappoport and Furche^44^

**Table:** 

F^−^ + CH_3_CH_2_I				Product branching fraction	
Collision energy		*k*_cap_	*k*	I^−^	FHI^−^
(K)	(eV)	(10^−9^ cm^3^ s^−1^)			
300	0.039	2.9	3.3(7)	0.98(1)	0.02(1)
400	0.052	2.7	3.4(7)	0.98(1)	0.02(1)
500	0.065	2.5	3.4(7)	0.98(1)	0.02(1)
600	0.078	2.4	3.3(7)	0.98(1)	0.02(1)

**Table:** 

F^−^ + CF_3_CH_2_I				Product branching fraction		
Collision energy		*k*_cap_	*k*	FHF^−^	I^−^	CF_3_CHI^−^
(K)	(eV)	(10^−9^ cm^3^ s^−1^)				
300	0.039	2.7	2.7(7)	0.93(2)	0.06(2)	0.01(1)
400	0.052	2.5	2.6(7)	0.93(2)	0.06(2)	0.01(1)
500	0.065	2.4	2.6(7)	0.90(2)	0.07(2)	0.03(2)

**Fig. 2:**
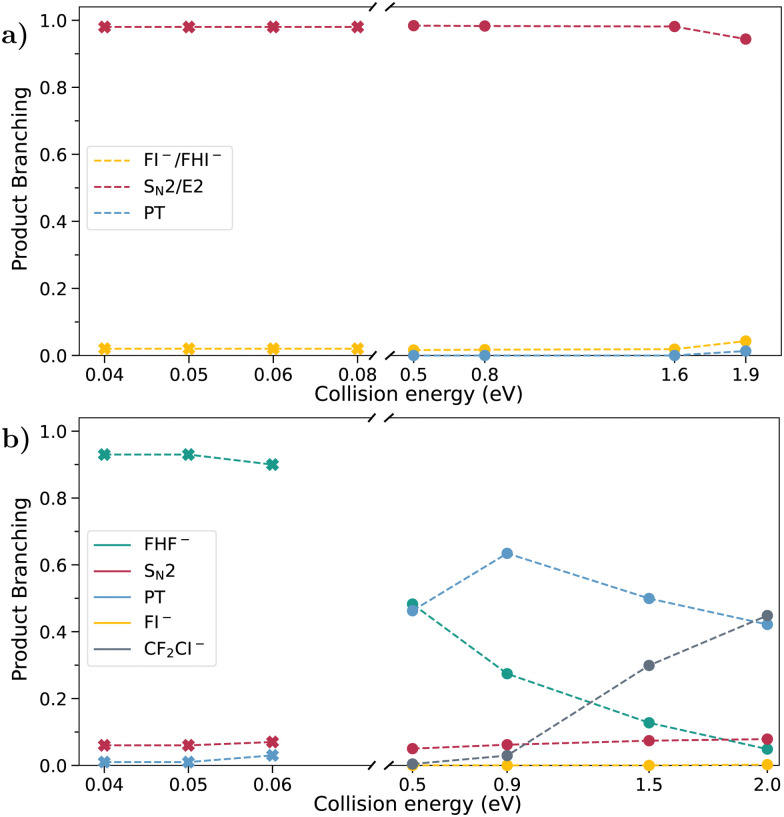
Product branching ratios for the different mechanisms in the reactions (a) F^−^ + CH_3_CH_2_I and (b) F^−^ + CF_3_CH_2_I. Data points in the lower collision energy range, marked by a cross, are from selected-ion flow tube measurements. Those in the higher energy range, marked by a point, are from the crossed-beam experiment.

The combined branching ratios for the low and high energy ranges are given in Fig. 2. Here, the crossed-beam imaging spectrometer provides the branching ratio for the collision energies from 0.5 to 2 eV. In the reaction of fluoride with iodoethane, iodide is by far the dominating product across the whole range, with only a small decrease at the highest measured collision energy. Both S_N_2 and E2 can lead to the formation of I^−^ in this reaction. From earlier theoretical calculations, we know E2 to be the more prevalent one, with over 80% contribution to iodide formation at 1.9 eV collision energy.^21^ In the similar reaction F^−^ + CH_3_CH_2_Cl the maximum contribution of S_N_2 amounts to 30% at 2.0 eV.^22^ The fluorination of the β-carbon, however, passivates the E2 pathway, leading to I^−^ being between 6% at the lowest and 8% at the highest collision energy of the overall product ions in the reaction with CF_3_CH_2_I.

A proton could be abstracted from both the α- or β-carbon. While the former is an endothermic process (0.80 eV) leading to the stable CH_3_CHI^−^, the latter leads to a subsequent E2 breakup and no CH_2_CH_2_I^−^ product is obtained in the computations.^22^ No product ion corresponding to proton transfer is detected in the SIFT experiment and at the lowest collision energy in the crossed-beam experiment. Proton transfer also only accounts for a fraction of product ions at the higher energies. In 1,1,1-trifluoro-2-iodoethane proton transfer is already observed in the SIFT measurements and becomes the dominant channel at 0.9 eV collision energy.

The halide abstraction channels, leading to FI^−^ in both reactions and FHI^−^ exclusively in iodoethane, are only a minor contribution to the products at all collision energies and only increase slightly above 1.5 eV. For iodoethane, both channels are summed up, because they are difficult to quantify separately with a suitable accuracy. In iodoethane, the exothermic (−2.23 eV) formation of FHI^−^ is observed both in the SIFT and crossed-beam measurements. However, the formation of FI^−^ is endothermic by 1.05 eV and is therefore not observed at the lower collision energies. The same is true for the reaction involving CF_3_CH_2_I, where the formation of FI^−^ is similarly endothermic (1.02 eV). FHI^−^, however, is not among the detected products, leading to the assumption that the additional hydrogen is abstracted from the β- rather than the α-carbon.

Apart from the above-discussed channels, we observe the formation of CF_2_CI^−^ in the reaction F^−^ + CF_3_CH_2_I. This channel is not open at lower collision energies due to its endothermicity by 0.98 eV but becomes increasingly important once energetically accessible.

One final reaction of interest in 1,1,1-trifluoro-2-iodoethane is the formation of FHF^−^. In the SIFT measurements, it is responsible for at least 90% of the product ions. At the lowest attainable collision energy in the crossed-beam experiment of 0.5 eV, it still accounts for 50% of the products. With increasing collision energy, however, it declines in significance.

In the following subsections the individual channels for both reactions, F^−^ + CH_3_CH_2_I and F^−^ + CF_3_CH_2_I, are discussed. For the former, only the S_N_2/E2 channel has enough statistics for meaningful analysis.

### 3.1 I^−^

**Fig. 3:**
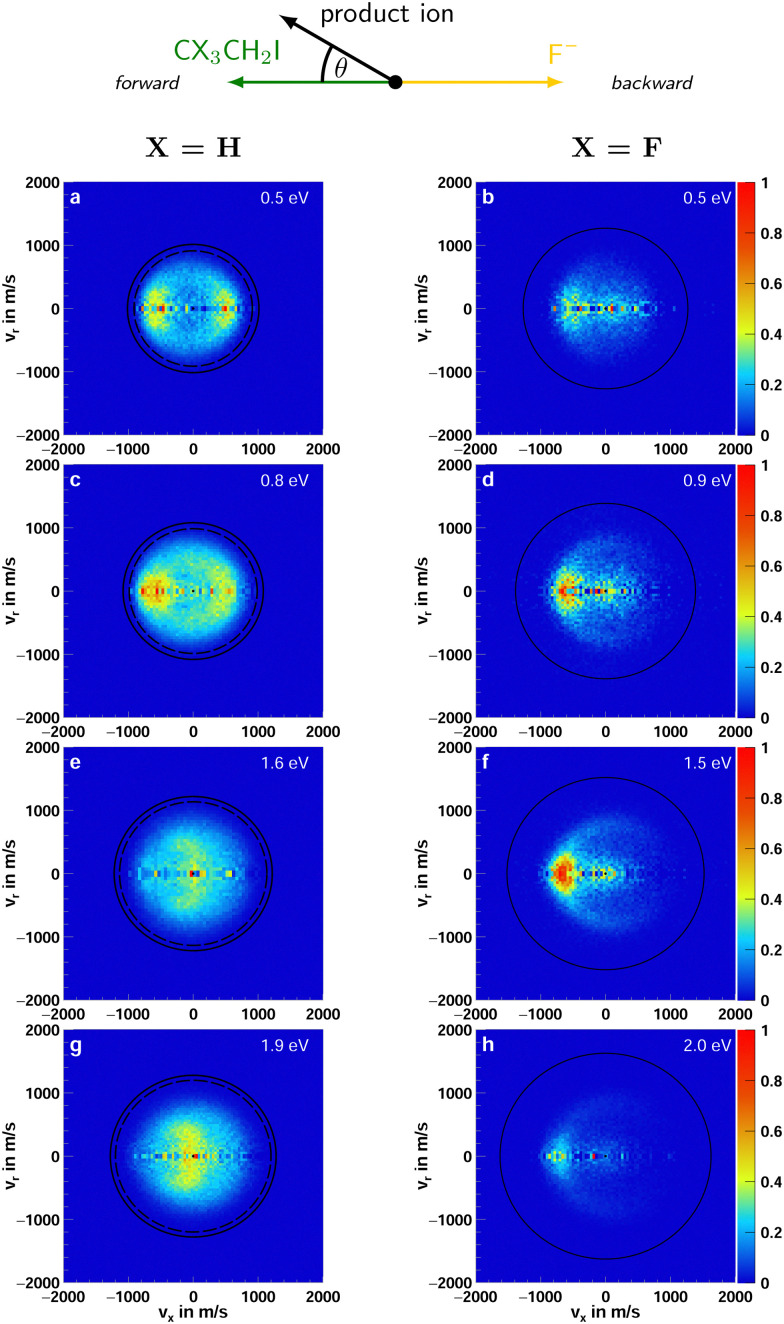
Accumulated product ion velocity distributions in the center of mass for I^−^ stemming from the reaction F^−^ + CH_3_CH_2_I (left column) and F^−^ + CF_3_CH_2_I (right column). The black circles represent the energetic limits resulting from the relative collision energy and the standard enthalpy of the reaction (kinematic cutoff). For the reaction with iodoethane, two sets of rings are presented. The inner ones depict the kinematic cutoffs for the E2 and the outer ones for S_N_2 reaction. In the case of the reaction with 1,1,1-trifluoro-2-iodoethane, the E2 reaction is passivated. Additionally, a schematic representation of the relative beam orientations is given at the top. The direction the initial neutral molecule traveled is defined as the forward and the one of the initial ion as the backward direction. The angle *θ* is the scattering angle of the product ion. The data used to produce (g) was part of a previous publication.^21^

**Fig. 4:**
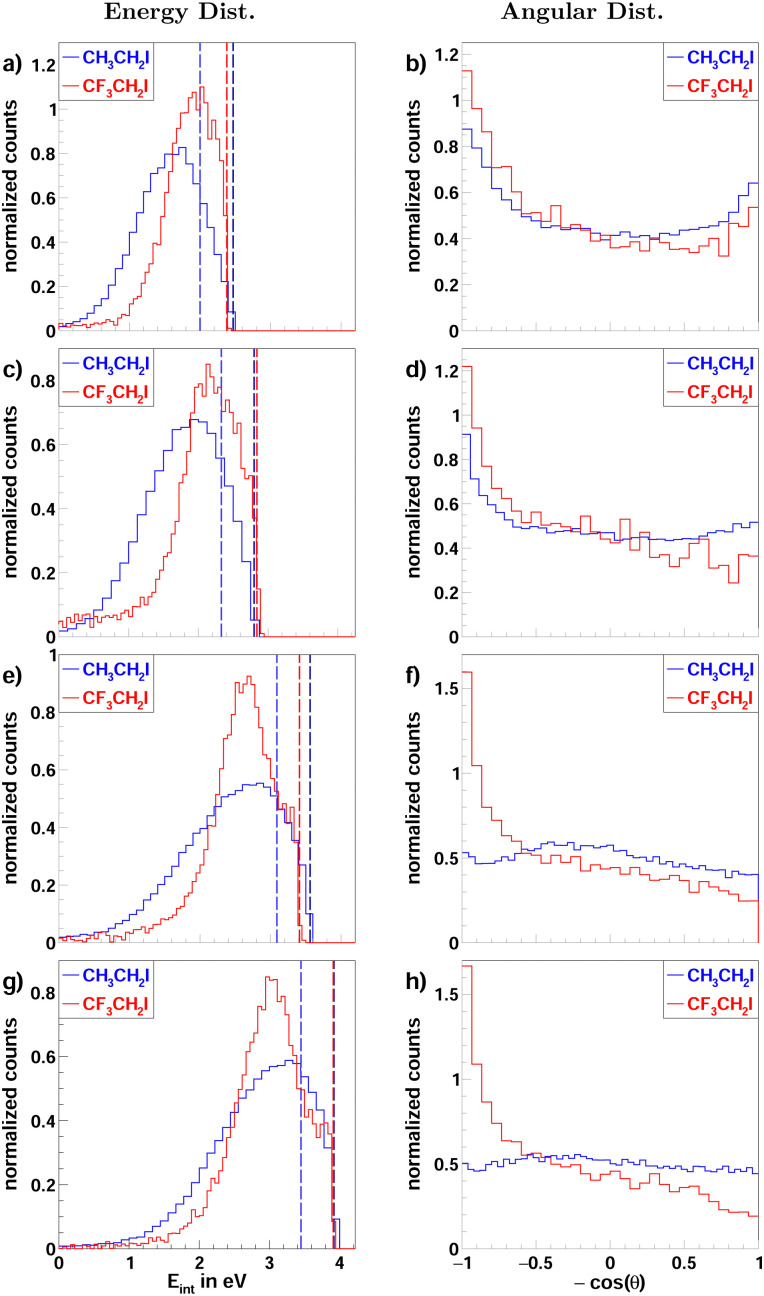
Normalized internal energy (left column) and angular (right column) distributions of the iodide product ions, from the reaction F^−^ + CH_3_CH_2_I (blue curves) and F^−^ + CF_3_CH_2_I (red curves). Each row depicts the distributions for one of the four measured collision energies 0.5/0.5 eV (a and b), 0.8/0.9 eV (c and d), 1.6/1.5 eV (e and f) and 1.9/2.0 eV (g and h). The superimposed lines in the internal energy distributions depict the maximum attainable energy calculated from the collision energy and the reaction enthalpy. The data for CH_3_CH_2_I used to produce (g and h) was part of a previous publication.^21^

Iodide as a product is observed in both F^−^ + CH_3_CH_2_I and F^−^ + CF_3_CH_2_I. As mentioned previously, in the reaction with iodoethane, I^−^ is primarily formed in the E2 type reaction. In the β-fluorinated molecule, only the S_N_2 reaction can lead to the formation of iodide. Fig. 3 presents the center-of-mass velocity distributions of a summation of iodide products for both reactions. The data used to produce Fig. 3g and 4g and h for the non-fluorinated reaction at 1.9 eV collision energy has been used in a previous publication.^21^

For iodoethane, a pronounced forward-backward symmetry is visible at lower collision energies (panels a–d in Fig. 3). This scattering feature has been assigned to an indirect, complex-mediated mechanism with large impact parameters in the E2-type reaction. Responsible for the symmetry of the differential cross-section is the conservation of angular momentum.^45–47^ This scattering behavior is reduced with increasing collision energy, while side-ways with partial forward scattering become more pronounced. The right column in Fig. 4 shows the angular distributions, where this progression is clearly visible. This pronounced side-ways with partial forward scattering has been ascribed to a direct stripping mechanism.^21^ Over all collision energies, we observe a high amount of isotropic scattering. The energy distributions of the products, depicted in the left column of Fig. 4, show that most of the energy is distributed into the neutral products and they peak close to the maximum available energy for the E2-reaction (first blue line). Part of the distribution extends beyond this though, implying that these ions stem from the S_N_2 pathway, which exhibits higher exothermicity. One has to consider here, however, the non-finite energy-uncertainty in the experiment, mainly stemming from the ion beam.

In the fluorinated reaction, all iodide products can be attributed to the S_N_2 channel. A somewhat similar forward-backward symmetry is visible in the differential cross-sections at the lowest collision energy. With its increase, the reaction becomes more direct, with the amount of forward scattered ions increasing. This type of scattering behavior is associated with a stripping-like mechanism, where the incoming fluoride strips off the CF_3_CH_2_ moiety and the iodide travels on in the same direction as the initial neutral CF_3_CH_2_I.^11,40,41^

### 3.2 FHF^−^

**Fig. 5:**
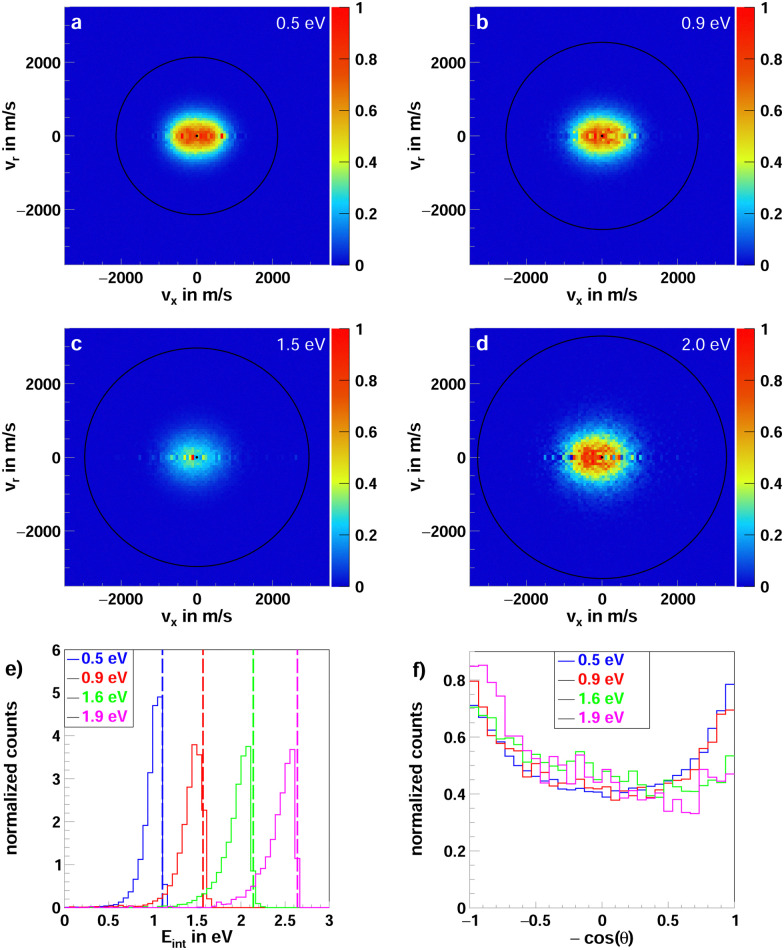
(a–d) Two-dimensional representation of the 3D center of mass velocity distributions of the product ion FHF^−^ from the reaction F^−^ + CF_3_CH_2_I at the four collision energies (a) 0.5 eV, (b) 0.9 eV, (c) 1.5 eV and (d) 2.0 eV. (e) Normalized internal energy distributions of the product ions. The superimposed lines depict the maximum available energy, calculated from the collision energy and the reaction enthalpy. (f) Normalized angular distributions of the product ions.

The velocity distributions for F^−^(FH) in the center-of-mass frame are shown in Fig. 5a–d for the collision energies 0.5, 0.9, 1.5, and 1.9 eV. The black rings show the highest possible product ion velocity (kinematic cutoff), determined by the collision energy and the standard enthalpy change. Over the whole energy range, the channel exhibits isotropic scattering into low velocities well below the kinematic cutoff. This, in turn, means high internal excitation of the products, which is apparent in Fig. 5e, where the internal energy distributions of the product ions are shown. The peak is close to the maximum available energy (colored lines), which means almost the entire energy is channeled into internal degrees of freedom rather than kinetic energy. This, together with the isotropic scattering, is indicative of the formation of a long-lived collision complex. The need for this complex formation can be appreciated by visualizing the reaction. The incoming F^−^ needs to both abstract a proton from the α-carbon and a fluorine from the β-carbon of 1,1,1-trifluoro-2-iodoethane, which involves breakage and formation of a multitude of bonds. Especially at the lower collision energies, an additional forward-backward symmetry, similar to the one in the iodide channel, is observed. This is indicative of an indirect mechanism involving large impact parameter events.^45–47^ At higher collision energies, this symmetry is lifted in favor of more forward scattering.

### 3.3 Proton transfer reaction

Although present in both un- and fluorinated reactions, proton transfer has almost no contribution to the former, and, due to low statistics there, cannot be meaningfully analyzed. In contrast, in the reaction with 1,1,1-trifluoro-2-iodoethane, it is one of the main pathways across the whole collision energy range (see Fig. 2).

**Fig. 6:**
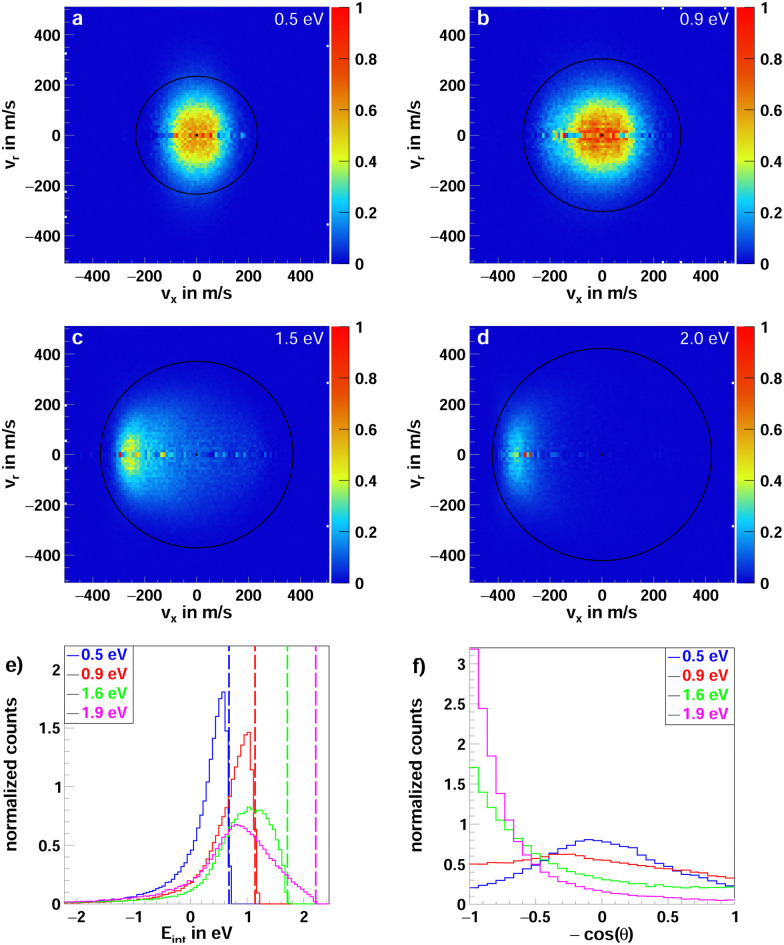
(a–d) Two-dimensional representation of the 3D center of mass velocity distributions for proton transfer from the reaction F^−^ + CF_3_CH_2_I at the four collision energies (a) 0.5 eV, (b) 0.9 eV, (c) 1.5 eV and (d) 2.0 eV. (e) Normalized internal energy distributions of the product ions. The superimposed lines depict the maximum attainable energy calculated from the collision energy and the reaction enthalpy. (f) Normalized angular distributions of the product ions.

In Fig. 6a–d, the velocity map images for the product ion CF_3_CHI^−^ stemming from the reaction F^−^ + CF_3_CH_2_I, for the four measured collision energies, 0.5, 0.9, 1.5 and 2.0 eV are shown. This reaction is known as proton transfer since the fluoride abstracts a proton from the neutral CF_3_CH_2_I, leaving the negatively charged CF_3_CHI^−^. The reaction is highly indirect at low collision energies, with most ions centered around zero velocity. With rising collision energy, the scattering behavior becomes more direct, with the ions ending up close to the kinematic cutoff scattered in the forward direction. This progression can additionally be seen in both the internal energy distribution (Fig. 6e) and angular distribution (Fig. 6f). In the former, the distributions for low collision energies peak close to the maximal available energy (colored vertical lines). With increasing collision energy, less is channeled into internal energies, leading to distributions peaking at lower energies. In the angular distributions, the isotropic nature of the indirect scattering can be observed at low collision energies. In contrast, the reaction becomes more direct, at higher energies, with mostly forward scattering.

The behavior observed in the differential cross-sections and the energy- and angular distributions can be interpreted by the formation of an intermediate collision complex at low energies, where the released energy is small enough to be efficiently distributed before the complex breaks up, leading to isotropically scattered ions with low velocity and highly internally excited products. At higher energies, the reaction can be described by a stripping-like mechanism. There, the incoming F^−^ abstracts a proton at large impact parameters, with too short interaction times to efficiently redistribute energy in internal degrees of freedom. This leads to the majority of ions traveling in the forward direction. Additionally, side-ways scattering can be recognized at low collision energies.

### 3.4 CF_2_CI^−^

**Fig. 7:**
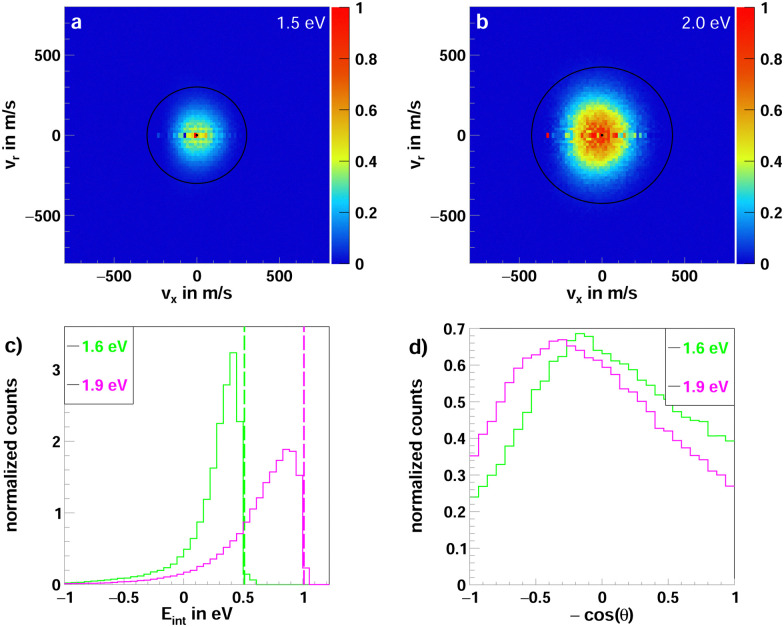
(a and b) Two-dimensional representation of the 3D center of mass velocity distributions of the product ion CF_2_CI^−^ from the reaction F^−^ + CF_3_CH_2_I at the two collision energies (a) 1.5 eV and (b) 2.0 eV. (c) Normalized internal energy distributions of the product ions. The superimposed lines depict the maximum attainable energy, calculated from the collision energy and the reaction enthalpy. (d) Normalized angular distributions of the product ions.

Due to the significant endothermicity of 0.98 eV of the reaction leading to CF_2_CI^−^ as the ionic product no signal is observed in the SIFT experiment and at the lowest collision energy (0.5 eV) of the crossed beam experiment. The opening of the channel is detected at 0.9 eV, as seen in Fig. 2. The reason for the discrepancy between the energy needed to overcome the endothermicity and the detection of products at a collision energy lower than the product relative energy stems from the energy width of the ion beam (around 150 meV FWHM). Once accessible, this channel becomes increasingly important. The scattering signature stays predominantly indirect across the whole collision energy range, which is to be expected due to the number of bonds that need to be broken and formed for the reaction to undergo. It is, therefore likely to form an intermediate complex, which is stable for at least a rotational period to lose any sense of initial velocity, scattering the products isotropically in space. Similar to the proton transfer reaction, we observe additional side-ways scattering, but with low kinetic energy (see Fig. 7).

## 4. Discussion

The reaction of fluoride with iodoethane leads almost exclusively to the formation of iodide as the ionic product, with only minor contributions of the two halide abstraction channels and proton transfer. The fluorination of the β-carbon in the neutral collision partner leads to a very different outcome. The E2 pathway is suppressed, and two additional channels involving the abstraction of fluorine open up. These new channels differ only in the abstraction of an additional proton, leading to the ionic products FHF^−^ and CF_2_CI^−^. They exhibit highly isotropic scattering across the whole collision energy range, implicating the formation of a long-lived collision complex. The need for this complex formation can be appreciated by their complexity, involving the breaking and forming of multiple bonds. A similar complex-mediated FHF^−^ formation has been found previously in the reactions of SF_6_^−^ with H_2_O and simple alcohols.^48^ In this channel, additional forward-backward symmetry can be observed in the differential-cross-sections. This indicates an indirect mechanism, which is reactive at large impact parameters. The symmetric structure along the collision axis results from the conservation of angular momentum.^45–47^ Contrary, the channel leading to CF_2_CI^−^ as the ionic product exhibits additional sideways scattering. This is usually attributed to a direct-stripping-like mechanism;^41^ here, however, these ions are still highly internally excited, as they exhibit low velocities far from the kinetic cutoff. A similar feature is observed in the proton transfer channel at low collision energies. With an increase in energy, this channel progresses to increased forward scattering, evolving from complete indirect to almost exclusively direct scattering behavior.

As mentioned in the results, proton transfer is almost non-existent in the reaction F^−^ + CH_3_CH_2_I. Only when the formation of CH_3_CHI^−^ is energetically accessible can some products be observed. This leads to the conclusion that CH_2_CH_2_I^−^ is only a transient species and leads to a nascent E2 breakup. Theoretically, a hydrogen shift along the carbon–carbon bond is feasible. Therefore, also CH_3_CHI^−^ could further breakup to form CH_2_CH_2_ and I^−^. In the reaction of fluoride with chloroethane (CH_3_CH_2_Cl), where full trajectory calculations were performed, such a shift was proposed to be possible.^22^ Further investigations of the trajectories, however, lead us to the conclusion that no E2-breakup happens on an α-hydrogen attack. The above conclusions explain the complete absence of proton transfer in the SIFT experiment and the only minor contribution of this channel at the higher collision energies.

Most strikingly, we see a reduction of iodide products with the introduction of the CF_3_-moiety. While this can largely be attributed to the inhibition of the E2 pathway, it also implies the decrease of S_N_2 reactivity. This was similarly observed in earlier studies.^24,49^ There, one contribution was reasoned to stem from the destabilization of the transition state due to the inductive effect of fluorine.^49^ Stationary point calculations, however, show no significant difference in energy of these in the reaction of F^−^ with CH_3_CH_2_I and CF_3_CH_2_I. The other detrimental influence on the S_N_2 reactivity was argued to come from electrostatic repulsion of the attacking nucleophile by the lone electron-pairs (*i.e.*, steric hindrance) of the fluorine atoms.^49^ We have used a similar argument in the study of the reaction F^−^ + (CH_3_)_3_CI, where we reasoned the crowding of the α-carbon center to be too extensive for the nucleophile to attack.^47^ Recently performed quasiclassical trajectory simulations on this system show, however, high intrinsic reactivity of the S_N_2 pathway when the E2 is artificially blocked.^9^ The experimentally observed reduced reactivity of S_N_2 is therefore reasoned to rather stem from the increased E2 reactivity than steric hindrance. A similar argument could be applied in the system F^−^ + CF_3_CH_2_I, where the opening of additional, highly reactive channels with the introduction of additional fluorine introduces competition to the otherwise reactive S_N_2 pathway. This is supported by the similarity of the van der Waals radii of fluorine (1.47 Å) and hydrogen (1.2 Å), further weakening the steric hindrance argument.

A direct comparison of the dynamics of the S_N_2 pathway in the reactions F^−^ + CH_3_CH_2_I and CF_3_CH_2_I is not possible due to the competition with E2 in haloalkanes. In the latter, however, a previously unidentified scattering feature can be observed, specifically, direct forward with partial high-angle scattering (see Fig. 3d, f and h). This is a previously unknown dynamic fingerprint for the S_N_2 reaction.^11^ Given that the energy barrier for the front-side attack is slightly decreased, this could possibly be a first experimental indication of this pathway.

## 5. Conclusion

For the reactions of F^−^ with CH_3_CH_2_I and CF_3_CH_2_I, respectively, rates and branching ratios, using a selected-ion-flow tube apparatus, and differential cross-sections, using a crossed-beam setup combined with velocity-map-imaging, were measured. Additionally, stationary points along the minimum reaction energy pathways were calculated using the CCSD(T)-F12a method. The dominant product in F^−^ + CH_3_CH_2_I is I^−^ at all measured collision energies, with only minor contributions of halide abstraction and proton transfer. Contrarily, in F^−^ + CF_3_CH_2_I, a plethora of additional channels are observed.

In the fluorinated reaction, the isolated S_N_2 signature shows a new forward scattering, stripping-like dynamic fingerprint. This might hint at the importance of front-side attack in the substituted species. The reactivity of the S_N_2 channel is, however, lower when compared to the non-fluorinated reaction due to the appearance of additional competing channels. These channels consist of the formation of FHF^−^, especially at lower collision energies, and CF_2_CI^−^, especially at higher energies. Both channels exhibit isotropic scattering with high internal excitation of the products. The former shows a known forward-backward symmetry in the differential cross-sections. A new sideways scattering mechanism coinciding with high internal excitation is observed in the latter. Accurate QCT calculations are needed to definitively assign the underlying mechanisms of the individual channels.

## Conflicts of interest

There are no conflicts to declare.

## Supplementary Material
